# We should not be complacent about our population-based public health response to the first influenza pandemic of the 21^st ^century

**DOI:** 10.1186/1471-2458-11-78

**Published:** 2011-02-03

**Authors:** Heath A Kelly, Patricia C Priest, Geoffry N Mercer, Gary K Dowse

**Affiliations:** 1 Victorian Infectious Diseases Reference Laboratory, Melbourne, Australia; 2 Department of Preventive and Social Medicine, University of Otago, Dunedin, New Zealand; 3 National Centre for Epidemiology and Population Health, Australian National University, Canberra, Australia; 4 Communicable Diseases Control Directorate, Department of Health Western Australia, Perth, Australia

## Abstract

**Background:**

More than a year after an influenza pandemic was declared in June 2009, the World Health Organization declared the pandemic to be over. Evaluations of the pandemic response are beginning to appear in the public domain.

**Discussion:**

We argue that, despite the enormous effort made to control the pandemic, it is now time to acknowledge that many of the population-based public health interventions may not have been well considered. Prior to the pandemic, there was limited scientific evidence to support border control measures. In particular no border screening measures would have detected prodromal or asymptomatic infections, and asymptomatic infections with pandemic influenza were common. School closures, when they were partial or of short duration, would not have interrupted spread of the virus in school-aged children, the group with the highest rate of infection worldwide. In most countries where they were available, neuraminidase inhibitors were not distributed quickly enough to have had an effect at the population level, although they will have benefited individuals, and prophylaxis within closed communities will have been effective. A pandemic specific vaccine will have protected the people who received it, although in most countries only a small minority was vaccinated, and often a small minority of those most at risk. The pandemic vaccine was generally not available early enough to have influenced the shape of the first pandemic wave and it is likely that any future pandemic vaccine manufactured using current technology will also be available too late, at least in one hemisphere.

**Summary:**

Border screening, school closure, widespread anti-viral prophylaxis and a pandemic-specific vaccine were unlikely to have been effective during a pandemic which was less severe than anticipated in the pandemic plans of many countries. These were cornerstones of the population-based public health response. Similar responses would be even less likely to be effective in a more severe pandemic. We agree with the recommendation from the World Health Organisation that pandemic preparedness plans need review.

## Background and Discussion

The World Health Organization (WHO) declared that spread of the newly recognised quadruple reassortant influenza A H1N1 virus satisfied the criteria for a pandemic on June 11, 2009,[1] although technically conditions for declaring a pandemic had been met some weeks earlier. The virus, generally referred to as pandemic influenza H1N1 2009 (pH1N1), had first been recognised in Mexico and the United States in late April 2009. More than a year later, WHO has declared the pandemic to be over and early assessments of the global response have commenced[2].

When the pandemic was declared, Dr Margaret Chan, the Director of WHO, advised member states to implement their pandemic plans [1] and health agencies, other government agencies and businesses worked hard to do this. In most countries it may be correct to conclude, as did an evaluation of the UK response, that the "pandemic and the response it generated have provided confirmation of the value of planning and preparedness"[3]. It is also true that the apparent success of the response in 2009 must not lead to complacency. We now know that the relatively low virulence of pH1N1 meant we did not need to have implemented effective responses to get a good outcome.

The response to the pandemic included clinical and public health measures. In developed countries, such as Australia, the clinical response was effective for those whose illnesses were serious[4]. Clinical care will very likely have reduced the number of deaths due to pandemic influenza [5], although the use of extra-corporeal membrane oxygenation was seen as a last resort and was not supported by the conclusions of a systematic review[6]. In developed and developing countries, the public health response focused on both the individual and the population. Individual responses promoted attention to personal hygiene, with an emphasis on cough hygiene and hand washing, which may not have been optimal [7], and the use of personal protective equipment for those considered to be at increased risk of infection[8]. Population-based public health responses to the pandemic focused on two major elements: non-pharmaceutical and pharmaceutical interventions. The former comprised border control and various elements of social distancing, while the latter focussed on anti-viral medication for treatment and/or prophylaxis, and the development of a strain-specific vaccine.

Australia used the pharmaceutical and non-pharmaceutical interventions detailed in its pandemic plan [9] in an effort to *delay *entry of the virus into the country, *contain *the virus to limited areas once it had entered the country, *sustain *a response when widespread community transmission had been established and to *protect *the vulnerable [10] - the latter being a new response phase formulated once it was realised that the pandemic was not associated with the high case fatality ratios that had been anticipated[11]. We use Australia's experience to draw attention to issues related to the public health population-based pandemic response. The scope of this perspective does not allow us to consider other categories of response.

Now is the time to acknowledge that a number of the strategies used in response to the 2009 pandemic could not control the spread of a novel influenza virus and their place in future pandemic response plans needs to be reconsidered in light of emerging new evidence. We examine four critical cornerstones of Australia's public health population-based response, namely border control, school closure (as an example of social distancing), the use of anti-viral medication and the development and use of a pandemic vaccine. We provide evidence from the pandemic experience in other countries to support our arguments.

### Border control

In a very different world, Australia successfully applied maritime quarantine to delay the entry of a pandemic H1N1 virus into the country in 1918 and 1919. This was in contrast to many of Australia's Pacific neighbours. For instance, the virus reached New Zealand in October 1918 but did not enter Australia until January of the following year. Estimated death rates in countries where the entry of the virus had been delayed were lower than rates where earlier entry was documented[12].

Prior to the 2009 pandemic, modelling studies had suggested a very limited role for border screening, providing an estimated delay of only 1-2 weeks without draconian measures that would be economically unacceptable in most countries[13]. A review of border control in Australia following the SARS epidemic pointed out the opportunity cost of screening. No case was identified despite 1.8 million passengers being screened, 794 referred for further evaluation and four identified as possible cases [14]. A similar lack of success was suggested in a review of the likely success of border control for influenza in other countries[15]. Indeed, it can be argued that prior to the pandemic there was only very limited scientific evidence for border control as an effective intervention. Despite this in 2009, as an island nation with history on its side, Australia, like many other (non-island) countries, embraced the concept.

Australia implemented a combination of approaches in an attempt to detect infected arriving passengers at international airports. These comprised notification of health status of passengers by airline staff (the pilot or crew identified passengers with respiratory symptoms), thermal scanning (infra-red cameras were installed in airport terminals), health declaration cards (the passenger reported current symptoms) and nurses at border entry points reviewed and tested passengers detected by one of these screening tools. In the Australian state of Queensland, although the number of passengers screened was not reported, and was likely to have been many tens of thousands, only four cases of confirmed pandemic influenza were found from 780 passengers identified by one or more of these border screening measures[16]. No cases were detected by similar screening at the busy international airport in Perth, Western Australia (unpublished data).

It has been suggested that Australia did well in its response to managing the pandemic, specifically in delaying establishment of community transmission[17]. During this time preparations were made to respond to a pandemic that was anticipated to result in many deaths. However, based on epidemiological and modelling evidence, we have demonstrated that community transmission was almost certainly established in the state of Victoria around the time the virus was first recognised in North and Central America in late April 2009 [18]. This followed one or more unrecognised silent importations, and spread of the virus in Australia came substantially from within its borders rather than from overseas. We now know that this is an entirely plausible scenario, given that a significant proportion of pH1N1 infections were afebrile[19] or entirely asymptomatic[20] and therefore impossible to detect at the border - or anywhere else.

This should not be surprising, as the finding that a high proportion of influenza infections are asymptomatic or afebrile was not new. Published experimental data from volunteer studies had previously shown that 33% of proven seasonal influenza infections were asymptomatic, but this varied by influenza type and subtype[21]. In particular, as few as 37% of experimental infections with influenza A(H1N1) were associated with fever recorded as >37.8°C, while 30% were completely asymptomatic[21]. Moreover, viral shedding in the pre-symptomatic phase of influenza infection has recently been confirmed to occur in approximately 1-8% of naturally acquired infections, in a study in which 14% of all influenza infections were asymptomatic and 31% of infections with influenza A (H1N1 or H3N2) did not have fever at the onset of other symptoms[22].

Prodromal, asymptomatic and afebrile infections cannot be detected by temperature measurement, one of the main components of border control, whether by thermal scanning or by core temperature measurement of symptomatic travellers[23]. Moreover, the proportion of afebrile or asymptomatic people is likely to be higher in infected travellers, as more severely unwell people will be less likely to travel. Thermal imaging is therefore even less likely to have been effective at the borders than in other places where more severely ill patients are seen[24]. Indeed, China used intensive thermal screening for pH1N1 at airports and had a positive detection rate of only 14 cases per million passengers screened[25].

The use of border control was evidently not based on a current understanding of influenza epidemiology and was not supported by modelling studies. In particular one modelling study, published two years before the identification of the current pandemic virus, showed that past pandemic patterns could not be adequately modelled without inclusion of asymptomatic infection (as well as varying degrees of pre-existing immunity) [26].

Nonetheless, an early evaluation of the 2009 pandemic, with limitations acknowledged by its authors, suggested that border screening may have led to delays of 7 to 12 days in the establishment of local transmission[27]. We accept that border screening will have detected a limited number of influenza cases, but suggest that many more cases will have been missed than were detected. In Australia, at least, it is likely that border screening was implemented after the virus had entered the country [18]. On balance, we conclude that border screening was as ineffective as it should have been expected to be.

### School closure

It is generally accepted that children, especially children of school age, are responsible for amplification of influenza epidemics[28]. An intervention targeting schools could therefore theoretically be effective in interrupting an epidemic. This assumption is supported by modelling studies, but only when all schools are closed early and remain closed for an unrealistically long period, up to the duration of the pandemic[29]. Modelling also shows that delay in closing schools, or partial closure of schools, are less effective interventions,[29] although, if school closures are timely, they may delay the peak and decrease the peak incidence of the epidemic [30].

As expected, the pH1N1 infection rate was high among school aged children[20,31]. Of the first 997 cases of confirmed pH1N1 infection in the state of Victoria in Australia, 67% were aged 5-17 years[32]. In Australia, school closure was intended to be associated with voluntary home quarantine. When a school - or class within a school - was closed, members of the class were asked to voluntarily quarantine themselves at home. This meant that parents of young children were frequently required to take time off work to care for children who would otherwise have been at school. Home quarantine has its own risks. We have recently shown that when an entire family was quarantined, the risk of secondary spread within households was increased by approximately 2.5-fold[33].

Moreover, compliance with other social distancing measures needed to have been effective for school closures themselves to have been effective. A survey in Western Australia of parents of school children whose schools were closed at some stage during the pandemic indicated that 74% of home-quarantined children participated in outside activities at least once during the nominal quarantine period, recording an average of 3.7 activities per child. Most commonly reported were attendances at sporting events, parks, beaches and stores, places where it is likely other children would be exposed[34]. Public documentation of school closure during the pH1N1 epidemic in Australia is minimal, but the policy in the early phase of the pH1N1 response was to close only those classes with confirmed cases, escalating to whole schools where multiple classes across different age groups were affected. In the state of Queensland only 2.8% of all schools were closed for short periods[16]. In Western Australia school closures were only for one week and sometimes involved only closure of specific classes[34]. Too limited in scope and time, these strategies could not have been effective in interrupting the spread of the pandemic.

On the other hand, experience in Japan confirmed the conclusions from modelling [30]. Early widespread school closures in a defined area were successful in delaying pandemic spread in that area, but when the schools were re-opened, pandemic spread resumed[35]. In Hong Kong closure of kindergartens, pre-schools and primary schools appeared to decrease the attack rate in children aged less than 12 years for the weeks of closure [36] but the effect on the final attack rate in school children is yet to be evaluated. Indeed, it has been argued that the potential benefit of closing schools during a pandemic must be balanced against the enormous social disruption that ensues[37]. Only where schools were closed early and remained closed would there have been any significant interruption of the spread of the pandemic.

### Neuraminidase inhibitors for treatment and prophylaxis

Countries around the world adopted different approaches to the use of neuraminidase inhibitors (NAIs) in their pandemic plans. In addition to treatment provisions, Australia opted for a stockpile of approximately 10 million courses of NAIs with the intention of implementing widespread prophylaxis, which has been shown in trials to be 58-84% effective in preventing laboratory proven influenza infection if given early following exposure[38]. However, even in the early phases of the response, when numbers of suspected and confirmed pH1N1 cases were low, those with responsibility for contact tracing were rapidly overwhelmed. The logistical difficulties of timely delivery of NAIs to those eligible for treatment or prophylaxis were such that it was likely only a minority received their medication in time for it to be effective. Lateness of NAI availability has been confirmed in a Victorian study of treatment doses. Oseltamivir was prescribed for only 207 (21%) of the first 1,000 confirmed cases. Of 690 cases confirmed not to have received oselatamivir, 670 were not eligible because more than 48 hours had elapsed since symptom onset (Unpublished data, James Fielding, epidemiologist, Victorian Infectious Diseases Reference Laboratory).

Other approaches to NAI distribution were used around the world, with varying effectiveness. For example, the UK National Health Service implemented an electronic checklist to allow patients rapid access to NAIs. Bypassing doctors and laboratory testing, this system aimed to speed up NAI availability. However, only 1932/16,560 (17%) of people who received NAIs using the electronic checklist subsequently tested positive for pH1N1[39].

On the other hand, in four outbreaks in Singapore military camps, when NAIs were able to be delivered effectively in conjunction with isolation of confirmed cases and quarantine of contacts, a beneficial effect could be demonstrated. These measures, which included ring prophylaxis with oseltamivir, resulted in a reduction of the infection rate in the outbreaks from 6.4% to 0.6%[40]. This study demonstrates the potential benefit of NAIs if available early in outbreaks, and when combined with social distancing. However extension of this strategy to large heterogeneous populations remains unproven and it may be feasible only in closed communities, such as boarding schools, military barracks and residential care facilities.

Another important consideration in setting out to provide mass treatment and prophylaxis with NAIs is the possibility of development of resistant strains. Surveillance studies during the first wave of the pandemic demonstrated a low frequency of resistance to oseltamivir and no reported resistance to zanamivir. Prior to 2007, it was rare to detect oseltamivir-resistant influenza strains in untreated patients, due to the compromised infectivity and transmissibility of many of the resistant mutants in the absence of drug pressure. But in 2007/2008 an oseltamivir-resistant seasonal A(H1N1) variant emerged that demonstrated viral fitness at least equivalent to the oseltamivir-susceptible strain. The resistant strain spread rapidly around the world and by 2009 had completely replaced the susceptible strain[41]. An oseltamivir-resistant pH1N1 virus might also retain viral fitness and subsequently spread throughout the community. Fortunately, to date, only a low frequency of oseltamivir-resistant pH1N1 strains have been identified[42].

An anti-viral stockpile without a well-developed logistic strategy and resourcing for effective early delivery for treatment of cases and prophylaxis of contacts is not an adequate plan for successful limitation of viral spread in a population, especially considering that the high proportion of cases with asymptomatic and mild infections will not be identified. Moreover, as we have seen with seasonal H1N1 viruses, resistance may develop to NAIs and a resistant virus may retain viral fitness allowing it to become widespread. This would render stockpiles useless. Revised pandemic plans should therefore consider limiting the use of NAIs to treatment of those with more severe influenza infection or medical conditions that make them more vulnerable to complications. Dependent on the availability of NAIs and access to appropriate medical care, treatment should be commenced early in the course of the illness. In Germany the median delay between symptom onset and antiviral treatment was significantly longer in fatal cases than non-fatal cases [43]. Prophylaxis should probably be reserved for closed communities, with any plan for wide-scale use of prophylactic NAIs dependent on a large workforce able to perform contact tracing and a detailed logistics plan for early delivery.

### A pandemic strain specific influenza vaccine

After China, Australia was the second country in the world to roll out a population-based pandemic vaccine program, with monovalent pandemic vaccine available by 30 September 2009[44]. The first wave of the pandemic in Australia had ended by this date. It was not expected that the vaccine would have been available in time to modify the first pandemic wave anywhere in the world. However, even in Australia, it was a case of 'too much too late'. An early estimate of 18% was made for population wide coverage for the vaccine [45].

Most pandemic vaccines in Australia were formulated as multi-dose vials. Given recommendations that the vial contents should be used or discarded within 24 hours of first use, wastage was expected with this formulation. It has been estimated that around 40% of pH1N1 vaccine doses delivered to Australian general practices may have been wasted[46]. There was also concern among some immunisation providers, and within the general community, that the multi-dose vials contained the preservative thiomersal, which had been phased out of paediatric vaccines, and that use of the vials potentially increased the risk of contamination, including with blood-borne viruses[47]. Such concerns, whether ill-founded or not, were likely to have impacted adversely on vaccine uptake, even in identified high risk groups [48]. While it may be reasonable to assume that vaccine uptake would have been higher if the disease had indeed been more severe, future pandemic plans need to include greater flexibility in vaccine purchasing and contracting arrangements, and refinement of vaccine delivery protocols and public messaging, in order to minimise wastage and optimise uptake [49].

The 21st century marks the first time pandemic-specific vaccines have been manufactured on a large scale. A preliminary report from Germany using the screening method estimated pandemic vaccine effectiveness for an adjuvanted pandemic vaccine of 97% in people aged 14-59 years[50]. A similar high level of protection has been reported for children in Canada[51], although a more modest effectiveness of 72% has subsequently been reported from a pooled case control analysis from a number of European countries[52]. While vaccines were effective in protecting individuals, population coverage in Australia and other countries was unlikely to have been sufficient for the vaccine to have modulated the spread of the pandemic virus. However some European countries, such as Germany, experienced a very modest first pandemic wave [43] and, had they achieved high coverage with pandemic vaccine, may have been able to modify pandemic virus transmission in the next influenza season. Nonetheless, the experience with pH1N1 suggests that a pandemic vaccine will always be too late, at least for one hemisphere, using current vaccine manufacturing technology.

## Summary

Control of pandemic influenza is a critical issue and one on which the world has already spent billions of dollars, both in planning and during the recent response to pH1N1. There are obvious lessons to be learnt from the first pandemic of the 21st century, a pandemic which was much less severe than many plans had anticipated[53]. If we think our response to this pandemic was adequate, we may be falsely reassured. A more severe pandemic may find us wanting. A mild pandemic may find us over reacting. However, with appropriate collection and analysis of data it should be possible to identify the severity of future pandemics early and to make a measured response[54]. The World Health Organization, governments and other agencies around the world are currently involved in reviews of the management of the pandemic[55]. It is vital that these reviews, while not diminishing the commitment and hard work of those who implemented the response plans in 2009, carefully assess the evidence base for those plans.

In addition, the widespread implications of the response to the pandemic - for policy makers, health professionals and the public - make it important for these reviews to be in the public domain. In Australia, where pandemic reviews are not yet in the public domain, there were examples where messages appeared to be mixed, and which confused both the public and healthcare professionals[56]. Partially closing some schools for short periods and not implementing other social distancing measures, such as cancelling public gatherings, is just one example.

Although we have provided examples from Australia, we believe our arguments will have relevance for many other countries. 'One size fits all', where authorities have only one response strategy for viruses with different infection rates and case fatality ratios, is not an appropriate response to pandemic preparedness. Revised pandemic plans should include different responses for different pandemic severities[57]. All areas of pandemic planning need to be re-examined, but perhaps by alternative processes to those that led to current plans. Certainly, new evidence about the practical difficulties and/or ineffectiveness of control measures, such as border control and school closures, needs to be considered seriously. The inadequacy of many plans has recently been publicly acknowledged by the head of the WHO's global influenza programme. Speaking at a United Kingdom Health Protection Agency conference on the international response to the H1N1 pandemic, Dr Sylvie Briand is reported to have said that the containment strategy during the last pandemic was 'not feasible' and that guidelines might have to be overhauled[58]. We believe this is sound advice.

## Competing interests

The authors declare that they have no competing interests.

## Authors' contributions

HK conceived the study and wrote the first and final drafts. PP revised and improved the first draft. All authors contributed to sequential drafts and reviewed and approved the final manuscript.

## Pre-publication history

The pre-publication history for this paper can be accessed here:

http://www.biomedcentral.com/1471-2458/11/78/prepub
